# Exploring barriers, needs, and facilitators for clinical and translational research in Oklahoma: A sequential mixed-methods study

**DOI:** 10.1017/cts.2025.10066

**Published:** 2025-06-18

**Authors:** Motolani E. Ogunsanya, Laura A. Beebe, Janis E. Campbell, Nicole Holmes, Timothy VanWagoner, Judith James

**Affiliations:** 1 Department of Family and Preventive Medicine, University of Oklahoma Health Sciences, Oklahoma City, OK, USA; 2 TSET Health Promotion Research Center, University of Oklahoma Health Sciences, Oklahoma City, OK, USA; 3 Department of Biostatistics and Epidemiology, University of Oklahoma Health Sciences, Oklahoma City, OK, USA; 4 Oklahoma Clinical & Translational Science Institute and Department of Medicine, University of Oklahoma Health Sciences, Oklahoma City, OK, USA; 5 Oklahoma Medical Research Foundation, Oklahoma City, OK, USA

**Keywords:** Clinical and translational research, barriers, needs assessment, Oklahoma, mixed-methods research

## Abstract

**Introduction:**

Clinical and translational research (CTR) plays a vital role in improving health outcomes, but its success relies heavily on institutional support, infrastructure, and workforce capacity. This study aimed to explore the barriers, needs, and facilitators to conducting CTR in Oklahoma, highlighting both the strengths and gaps within the research ecosystem.

**Methods:**

A sequential, descriptive mixed-methods design was employed, combining survey data (*n* = 164) with four qualitative focus groups (*n* = 23 total participants). The survey assessed research infrastructure, funding, and workforce needs, while the focus groups explored researchers’ lived experiences and institutional challenges. Mixed-methods meta-inference approaches, such as convergence, complementarity, and explanatory integration, were used to identify overlapping and distinct patterns across data strands.

**Results:**

Key barriers included lack of protected research time (23.9%), limited pilot funding (15.3%), and administrative hurdles such as IRB delays. Researchers expressed a strong need for centralized tools to support networking, scientific writing, and data access. Qualitative findings revealed additional needs, such as bridge funding and mentorship, not fully captured in the survey. Facilitators included Oklahoma Shared Clinical and Translational Resources (OSCTR)-supported professional development and mentoring programs, though participants noted a heavy reliance on OSCTR as the primary support source, with few decentralized alternatives.

**Conclusions:**

While CTR infrastructure in Oklahoma has expanded, critical gaps remain in mentorship, data access, and institutional support. To build a more resilient and inclusive research environment, stakeholders should consider investing in decentralized systems, bridge funding, structured mentorship, and collaborative tools tailored to the state’s rural, tribal, and academic diversity. These findings may inform policy and strategic planning in Oklahoma and other underserved regions aiming to strengthen CTR capacity.

## Introduction

Clinical and translational research (CTR) is pivotal in advancing medical knowledge and improving health outcomes by bridging the gap between basic scientific discoveries and their application in clinical settings. It ensures that research findings are translated into medical practices and therapies that benefit patients [1]. CTR encompasses a range of activities, from basic science and clinical trials to implementation science, aimed at driving meaningful improvements in healthcare delivery. However, its success is shaped by more than scientific discovery alone; it depends heavily on the research environment, including infrastructure, funding, workforce capacity, and institutional support [2]. These structural components are essential in settings with persistent health disparities, where robust CTR systems help ensure that underrepresented populations benefit from research advancements.

Oklahoma presents a compelling case for examining CTR capacity. The state consistently ranks among the lowest nationally across multiple health indicators and experiences some of the most pronounced disparities in the country [3,4]. According to the United Health Foundation, out of 50 states in the United States, Oklahoma ranked 45^th^ in overall health outcomes in 2022, and near the bottom in obesity (48^th^), diabetes (46^th^), smoking (49^th^), and cardiovascular deaths (48^th^) [3–6]. These challenges extend beyond individual behavior or access to care; they reflect systemic issues, including limited capacity to generate, translate, and apply health research locally.

In response, the National Institutes of Health (NIH) supported the creation of the Oklahoma Shared Clinical and Translational Resources (OSCTR) in 2013 to build CTR capacity statewide. Housed at the University of Oklahoma Health Sciences (OUHS), OSCTR is a collaboration of 15 institutional and community partners, including the Cherokee Nation, Southern Plains Tribal Health Board, and the Oklahoma City Veteran Affairs, working to expand research infrastructure, training, and mentorship with an explicit focus on underserved and rural populations [7,8]. Over the past decade, OSCTR has made considerable strides in providing pilot funding, professional development, and support services to researchers across the state.

Despite these efforts, persistent gaps remain. Many researchers, especially those in rural and tribal regions, still face limited access to data, underdeveloped mentorship structures, and fragmented infrastructure [3,7]. These shortcomings may explain why gains in health outcomes have not kept pace with infrastructure investments. Furthermore, past evaluations of CTR capacity in Oklahoma have primarily relied on quantitative indicators, such as grant counts and enrollment rates [3,7], which do not capture researchers’ lived experiences or institutional barriers.

To address this gap, we conducted a sequential, descriptive mixed-methods needs assessment, integrating survey data with qualitative input from CTR investigators and community partners. This design enables a more nuanced understanding of the barriers, facilitators, and unmet needs shaping research capacity across Oklahoma. By examining both the measurable and the experiential aspects of CTR, this study aims to inform a more equitable, effective, and sustainable research infrastructure across OSCTR and its partners [9,10]. As OSCTR enters its second decade, these findings can guide strategic investments, strengthen interdisciplinary collaboration, and inform the redesign of support systems to better serve Oklahoma’s unique research ecosystem. Ultimately, this work aims to ensure that CTR investments translate into meaningful improvements in health outcomes, particularly for the state’s most underserved communities, and also offers a model for other states facing similar challenges.

## Methods

### Study design

Using a sequential, descriptive exploratory mixed-methods design, this study examined the barriers, needs, and facilitators to conducting CTR among active CTR researchers in Oklahoma. This design was chosen because it provides a comprehensive understanding of the phenomenon of interest [11]. We began with a quantitative phase to gather data on research infrastructure, funding levels, and other objective metrics related to CTR across the state. This phase provided a broad overview of the research environment and helped identify initial trends and patterns. Subsequently, we conducted a qualitative phase, comprising focus groups with active CTR researchers, to explore their subjective experiences. By integrating these two phases, we aimed to enhance the validity and richness of our findings, capturing both the breadth and depth of CTR landscape in the state. This design also supported an iterative and reflexive research process, enabling the refinement and exploration of emerging themes throughout the study.

### Study setting

The OSCTR initiative, launched with a $20.3 million grant from the NIH in September 2013, represents a significant collaborative effort led by OUHS [7]. The initiative includes partnerships with a wide range of institutions across Oklahoma, such as the Oklahoma Medical Research Foundation, the University of Oklahoma’s Norman (OU Norman) and Tulsa campuses (OUHS Tulsa), the Veterans Administration, Oklahoma State University and its Health Sciences Center, among others. These partnerships are instrumental in advancing CTR programs in Oklahoma, offering essential research support services, and facilitating faculty development in translational research methodologies. In line with its overarching goals, OSCTR has initiated a comprehensive needs assessment to ensure the alignment of its services with the current requirements of CTR researchers and partner institutions.

### Target population

**Figure 1.**
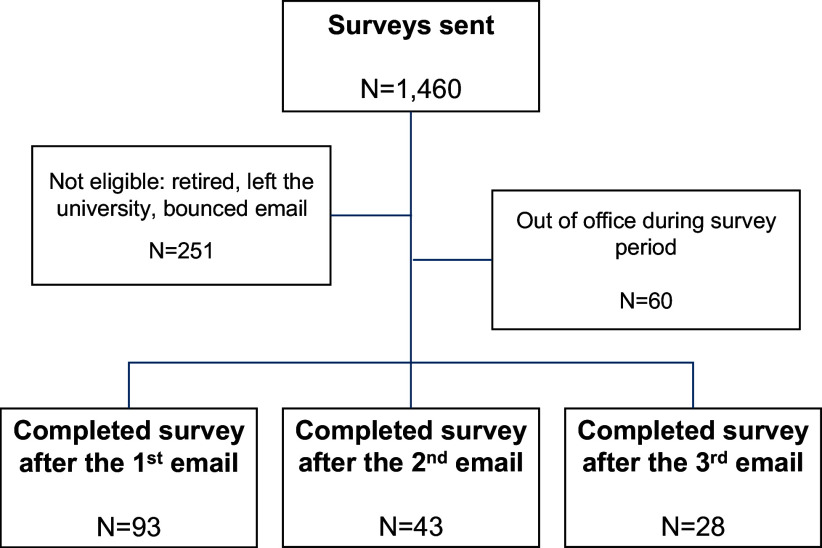
Sampling flowchart.

The target population comprised active CTR researchers identified from the IRB protocol management system at OUHS. Individuals listed as Principal Investigators or Co-Investigators with ongoing IRB-approved protocols were included in the sampling frame. The initial sampling frame consisted of 1,460 unique investigators with active IRB protocols. After the initial survey invitation email, 251 investigators were identified as “ineligible” because they had retired or left the university. An additional 60 investigators were inaccessible during the survey period based on out-of-office email responses. This resulted in a final sample of 1,149 eligible participants. After three invitations to participate in the survey, 164 responses were received, yielding a response rate of 14.3% (see Fig. 1 for sampling flowchart).

### Data collection

Data collection occurred in two phases: phases one and two. In phase one, the exploratory stage, barriers, needs, and facilitators were initially identified through a web-based survey (*n* = 164) (see Appendix A). The survey was modeled after several instruments, including the 2012 OSCTR survey [7], the Advance-CTR (Rhode Island) [12], and the STRiDE needs assessment (Delaware) [13]. The initial draft was reviewed by OSCTR leadership, and revisions were made based on their feedback. The survey was then pilot-tested with a small group of investigators to improve relevance, ensure clarity of questions, and reduce the overall questionnaire length. The final survey included the following sections: description of research activities, barriers, needs, and facilitators to CTR, awareness of OSCTR services and resources, and demographics (age, gender, current position/academic rank, degrees held, primary affiliation, types of research, manuscript authorship activity). A final question asked if participants would be interested in participating in a follow-up interview. The survey was administered online using REDCaP [14]. An initial email invitation containing the survey link was distributed in February 2019, with four follow-up reminders sent over the next four weeks before the survey closed in March 2019. Descriptive statistics were analyzed using SPSS v25.

In phase two, the explanatory phase, findings from the survey were further explored through four qualitative focus groups involving 23 participants randomly selected from phase one. These focus groups were conducted between 2020 and 2022. The extended gap between the survey and the focus groups was due to the onset of the COVID-19 pandemic, which delayed both in-person and virtual data collection efforts during that time.

### Measures

#### Quantitative measures

**Description of research activities.** This was assessed by asking participants to indicate the types of research they conduct: clinical, basic, public health, education, community-based participatory, health services, dissemination and implementation, methodological, engineering, translational, and therapeutic development research.

**Demographics.** Participants provided demographic information, including gender, current position/academic rank, degrees held, primary affiliation, and types of research conducted. Information on manuscript submissions was gathered, such as whether participants had submitted manuscripts as lead, senior, or corresponding authors in the past year.

**Barriers.** To assess barriers to conducting CTR, participants rated the extent to which they perceived limited access to various research supports as impediments. These supports included grant administration, regulatory issues, conducting research, pilot project funding, and proposal development, and others. A total of 16 barriers were included, with responses captured on a scale from “Not at all” to “A great deal,” along with an option for “N/A.”

**Needs.** This was assessed by evaluating respondents’ anticipated utilization of various research support resources and services over the next 6 months. This assessment included inquiries about the likelihood of utilizing different tools and assistance, such as web-based research networking, online tutorials, individual assistance with research services, support with regulatory processes, participant recruitment, clinical testing equipment, data analysis expertise, and study design.

**Facilitators.** Facilitators were assessed by asking participants which services or resources provided by OSCTR’s Professional Development Core contributed most to advancing their CTR efforts. Options included scientific writing, bench-to-bedside collaboration, mentoring practices, data security, and access to specialized tools such as bioinformatics and mHealth technologies.

**Overall Satisfaction.** A one-item 5-point Likert scale (ranging from “Not at all” to “Extremely”) assessed participants’ satisfaction with the institution’s overall efforts at supporting CTR.

#### Qualitative measures

Focus group interviews were conducted in person by MEO, assisted by NH, and facilitated using a semi-structured interview guide developed by the study team to capture rich qualitative data aligned with the study goals. The guide included open-ended questions and specific probes to allow for in-depth exploration of barriers, needs, and facilitators not fully captured by the survey (see Appendix B). Key questions focused on barriers to CTR, such as challenges with resource identification, regulatory processes, protected time, professional collaboration, and institutional support. Additional questions explored training, funding, and professional development needs. Facilitators were examined to understand effective strategies for overcoming obstacles and meeting research needs. Focus groups also addressed potential solutions and implementation strategies to generate actionable strategies. All sessions were audio recorded and supplemented with interviewer notes. Interviews ranged 57–76 minutes and were professionally transcribed verbatim. After checking for accuracy, MEO coded each interview line-by-line and developed a coding framework using Atlas.ti v9. Audio transcripts were content-analyzed to create inductive categories, and generated codes were combined to form themes, which were labeled based on their order and frequency. To ensure reliability, additional team members independently tested the coding framework by comparing transcripts to identified themes. The themes were extracted through coding, developed from combined recordings and notes, and reviewed by the investigators. An audit trail was maintained throughout, and peer debriefing was conducted to enhance credibility. Final findings were presented to participants for feedback to enrich interpretation. The study was approved by the University of Oklahoma Institutional Review Board (IRB #9840), and informed consent was obtained from all participants.

#### Mixed methods integration

**Figure 2.**
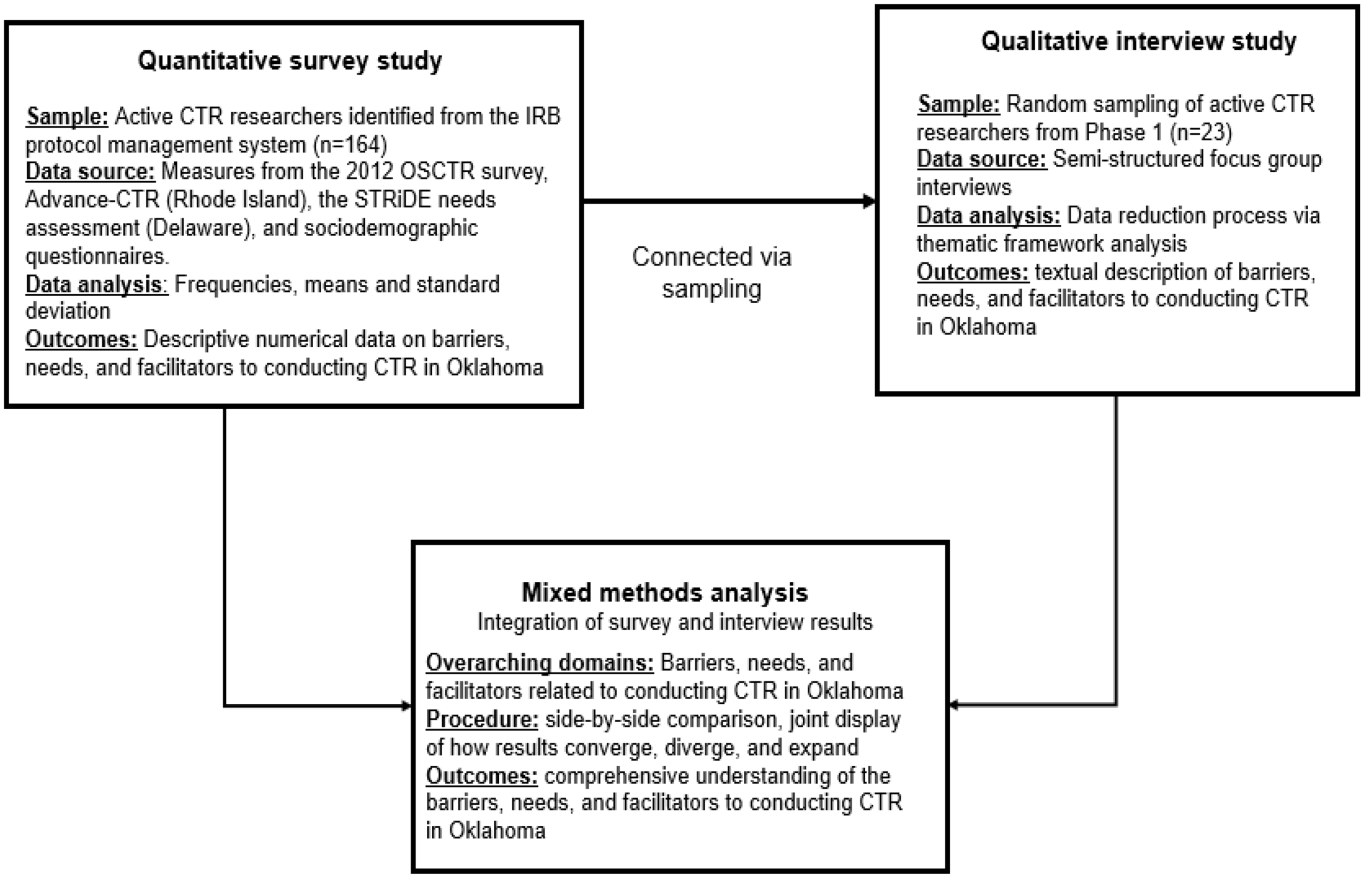
Sequential mixed methods design of the barriers, needs, and facilitators of clinical and translational research in Oklahoma. CTR = clinical and translational research; OSCTR = Oklahoma Shared Clinical and Translational Resources.

Integrating qualitative and quantitative data allowed for a comprehensive understanding of the barriers, needs, and facilitators in conducting CTR in Oklahoma. By combining these approaches, the study leveraged numerical data to identify patterns and trends. In contrast, qualitative data provided the underlying experiences and explanations, enhancing the overall depth and impact of the findings. To facilitate alignment across methods, themes and topics from the survey informed the development of the interview guide, allowing for consistency in data collection. Findings from both phases were then merged to explore convergence, divergence, triangulation, complementarity, and expansion across data types [15]. This integration enabled a robust and nuanced interpretation of the findings, combining statistical patterns with lived experiences to inform future CTR infrastructure improvements (see Fig. 2).

## Results

Participant Characteristics. A total of 164 researchers participated in the web-based survey, and 23 were included in the qualitative study. Most survey respondents were female (*n* = 84, 51.2%), with a slightly higher proportion of females in the qualitative group (*n* = 14, 60.9%). Most survey participants identified Medicine as their primary affiliation (*n* = 102, 64.2%). In contrast, the qualitative sample showed more diversity in affiliation, with Medicine (*n* = 9, 39.1%) being the most common, followed by Public Health (*n* = 4, 17.4%) and other disciplines. Regarding academic ranking, Assistant Professors were the most represented in the survey (*n* = 51, 31.1%) and the qualitative interviews (*n* = 11, 47.8%). A majority of participants held a PhD degree (survey: *n* = 82, 35.0%; qualitative: *n* = 12, 52.2%), and more than 70% of participants in both groups reported engagement in clinical or patient-oriented research (survey: *n* = 120, 73.2%; qualitative: *n* = 17, 73.9%). Table 1 presents the descriptive characteristics of all study participants.

**Table 1.** Quantitative characteristics for the overall sample and for the qualitative data participants

Variables	Quantitative survey study (*n* = 164)	Qualitative interview study (*n* = 23)
Gender (%)		
Female	84 (51.2)	14 (60.9)
Male	68 (41.5)	9 (39.1)
Academic rank or position (%)		
Assistant professor	51 (31.1)	11 (47.8)
Associate professor	44 (26.8)	8 (34.8)
Full professor	39 (23.8)	2 (8.7)
Postdoc/clinical fellow	6 (3.7)	–
Staff	11 (6.7)	2 (8.7)
Instructor	1 (0.6)	–
Other (graduate student, adjunct faculty)	7 (4.3)	–
Degrees held (%)		
PhD	82 (35.0)	12 (52.2)
MD	63 (26.9)	9 (31.1)
MS	37 (15.8)	4 (17.4)
MPH	20 (8.5)	–
Other	31 (13.2)	3 (12.7)
Primary affiliation (%)		
Medicine	102 (64.2)	9 (39.1)
Public Health	18 (11.3)	4 (17.4)
Pharmacy	10 (6.3)	3 (13.0)
Allied health	10 (6.3)	2 (8.7)
Nursing	9 (5.7)	1 (4.3)
Dentistry	4 (2.5)	–
Other	11 (6.9)	3 (13.0)
Type of research		
Clinical (or patient-oriented) research	120 (73.2)	17 (73.9)
Basic Research	55 (33.5)	8 (34.8)
Public or population health research	44 (26.8)	8 (34.8)
Education research	37 (22.6)	6 (26.1)
Community-based participatory research	22 (13.4)	3 (13.0)
Health services research	21 (12.8)	3 (13.0)
Dissemination and implementation research	20 (12.2)	2 (8.7)
Methodological research	7 (4.3)	–
Engineering research	2 (1.2)	–
Other (Translational, therapeutic development)	4 (2.4)	–

**Figure 3.**
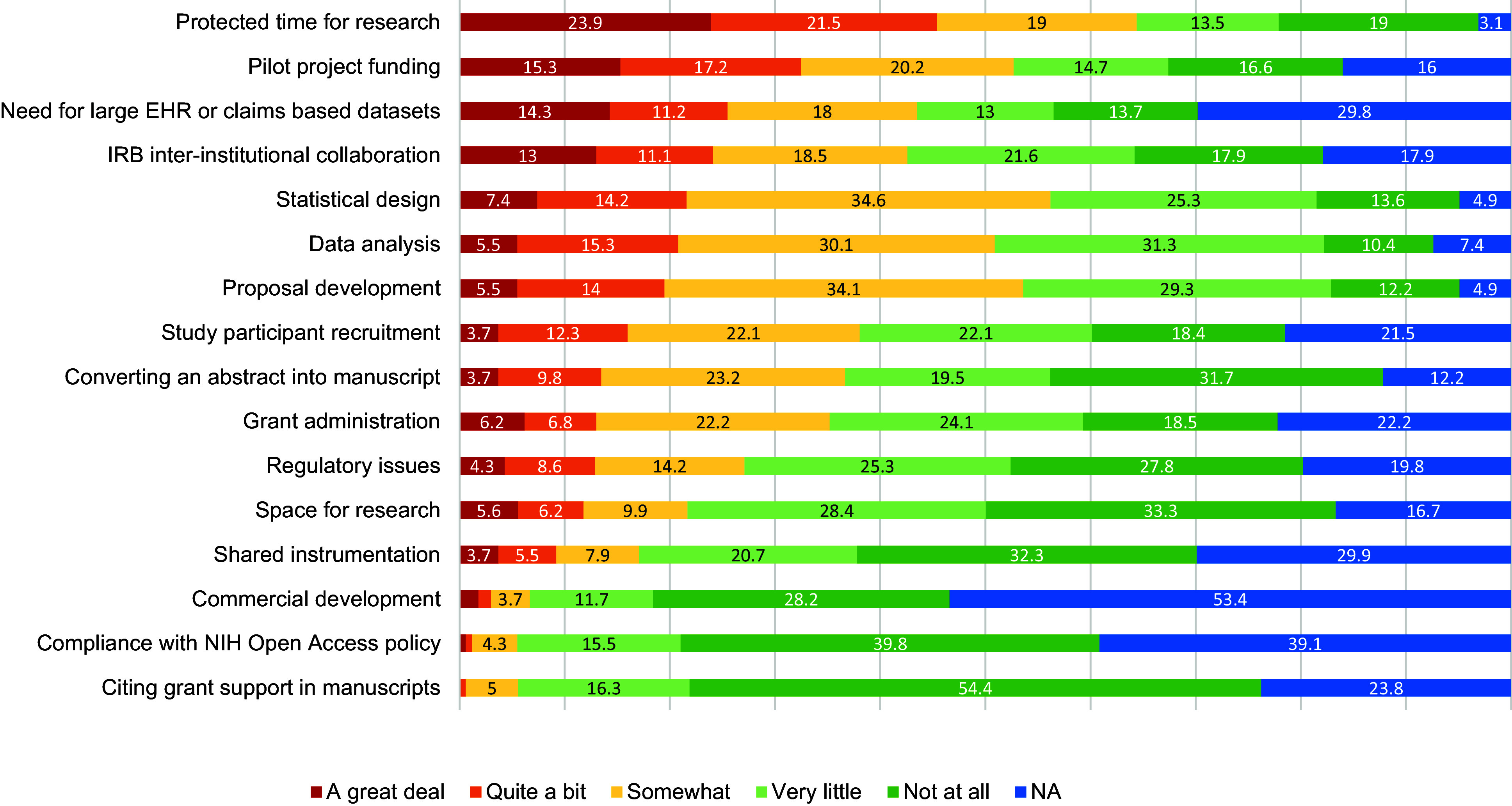
Barriers to clinical and translational research. Participants were asked: “In the past year, to what extent have you experienced the following barriers or challenges?” (See figure above). EHR = Electronic Health Record; IRB = Institutional Review Board.

**Figure 4.**
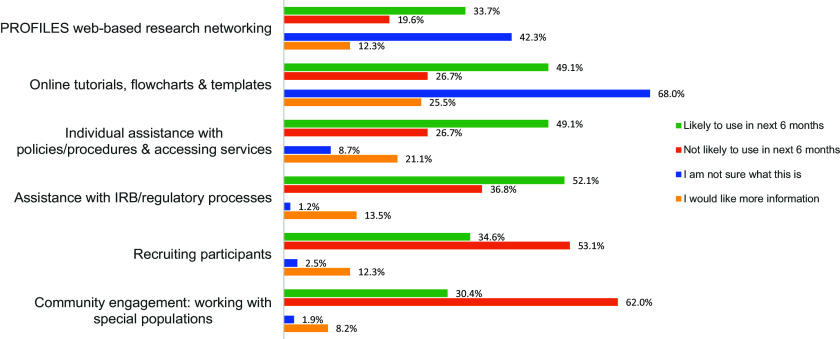
Clinical and translational research (CTR) needs. The Oklahoma Shared Clinical and Translational Resources aims to provide a wide range of services to support CTR. Participants were asked: “Please rate the level of interest in using these services.” (See figure above.)

**Figure 5.**
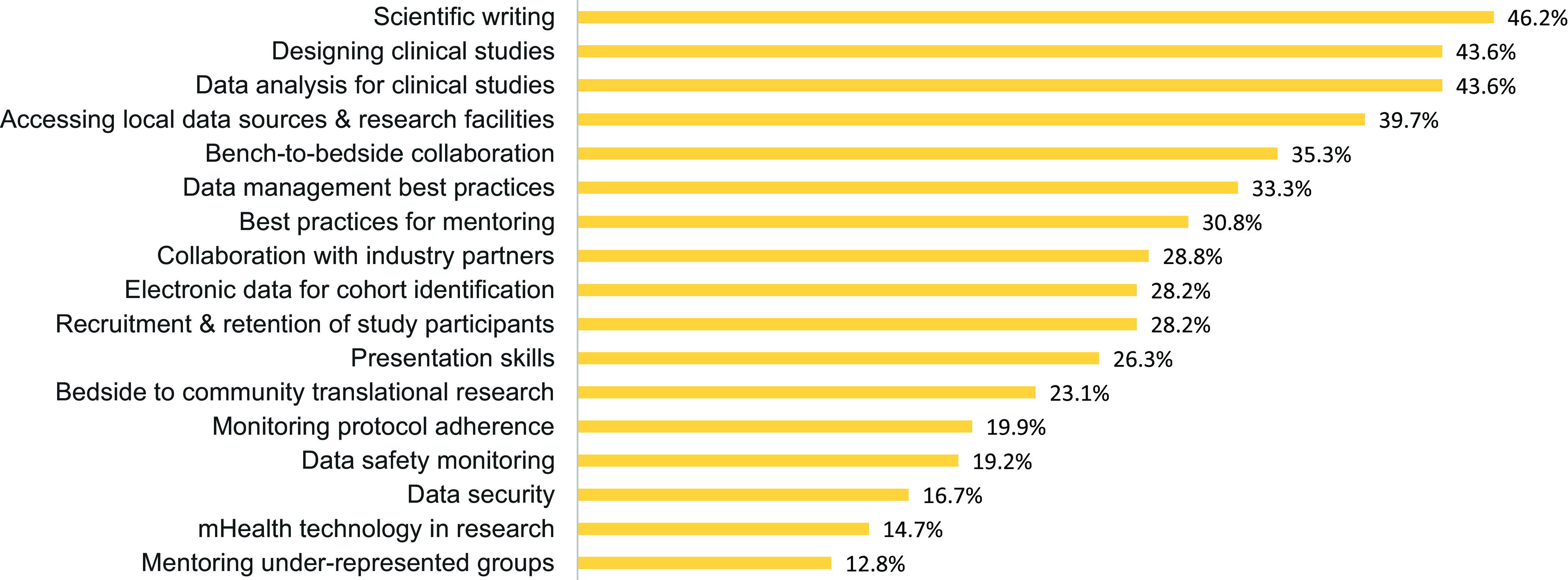
Facilitators. The Oklahoma Shared Clinical and Translational Resources (OSCTR) Professional Development Core offers educational opportunities to enhance the clinical and translational research (CTR) capabilities of investigators. Participants were asked to identify which of the following OSCTR-provided CTR-related activities has most helped them advance their research.

**Figure 6.**
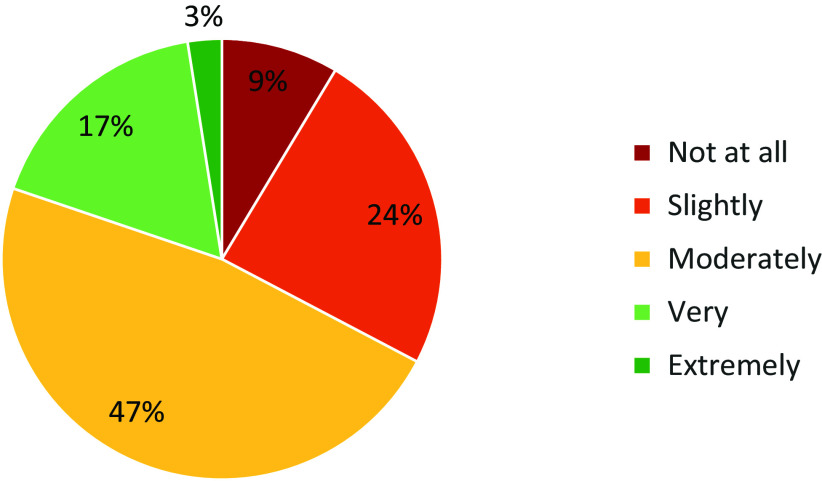
Overall satisfaction. How satisfied are you with your institution’s overall efforts at supporting clinical and translational research?

Barriers, Needs, Facilitators, and Overall Satisfaction in CTR. Notably, “Protected time for research” and “Pilot project funding” were identified as significant barriers, with over 15% of respondents indicating these as “a great deal” of a barrier. Challenges like IRB inter-institutional collaboration, data analysis, and proposal development also emerged as notable concerns. Regarding needs, there was strong interest in “Online tutorials, flowcharts & templates” and “Individual assistance with policies/procedures,” with nearly 50% of participants indicating they were likely to use these resources within the next 6 months. In contrast, services like “PROFILES web-based research networking,” received lower levels of awareness or interest, as suggested by the high proportion of respondents who were unsure about their potential use. Among the facilitators, “Scientific writing” (46.2%), “Designing clinical studies” (43.6%), and “Data analysis for clinical studies” (43.6%) were identified as the top activities that supported researchers’ work. Other areas of interest included “Accessing local data sources & research facilities” (39.7%) and “Bench-to-bedside collaboration” (35.3%). Finally, over 50% of respondents (67% total) were moderately-extremely satisfied with the institution’s support for CTR. However, about one-third expressed lower levels of satisfaction (“Slightly” or “Not at all”), pointing to potential areas for improvement in the institution’s support mechanisms. See Figs. 3–6 for a summary of the findings.

Mixed Methods Integration. Quantitative and qualitative data were integrated to better understand the barriers, needs, and facilitators in conducting CTR. This approach leveraged the strengths of both methods; quantitative data identified key trends and patterns, while qualitative data provided the context, depth, and explanations necessary to interpret those findings meaningfully. By triangulating results across diverse data sources, our study aimed to provide a comprehensive assessment of the factors shaping the CTR landscape and offer actionable recommendations to strengthen research capacity and collaboration across the state [10]. Table 2 contains a summary of these findings.

**Table 2.** Mixed-methods meta-inference of barriers, needs, and facilitators in clinical and translational research

Categories	Quantitative results	Qualitative quotes	Mixed-methods meta-inference
**Barriers**			
Protected time for research	23.9% reported this as “a great deal” of a barrier; 21.5% as “quite a bit.”	*“Securing protected time is a major challenge, especially when prioritizing patient care. While I have a collaborator who helps share the workload, this isn’t feasible for everyone. We need a system that values smaller contributions, recognizing that even 10% of dedicated input can be significant. Without such a structure, many clinicians feel they can’t engage in research, resulting in missed opportunities to harness valuable expertise and drive meaningful progress.” – Participant 3, focus group (FG) 1*	**Convergence:** Both data types indicated that the lack of protected research time is a substantial barrier, reinforcing the need for institutional change.
Pilot project funding	15.3% indicated this as “a great deal” of a barrier; 17.2% as “quite a bit.”	*“The barrier is around money because translational research usually involves teams… sometimes it’s such overwhelming obstacles just to try to put the team in place.” – Participant 13, FG 2*	**Complementarity:** The qualitative data provided context to the quantitative findings, highlighting that funding difficulties are tied to team-based research efforts.
Administrative & regulatory issues	13% reported “IRB and inter-institutional collaboration” as “a great deal” of a barrier; 11.1% as “quite a bit.”	*“Right now, ORA and IRB are slowing down everything. I don’t know what’s going on right now, but it’s really bad.” – Participant 6, FG 1*	**Explanatory:** Qualitative data explained the quantitative findings, showing that IRB delays and complexity significantly contributed to administrative barriers.
Data access	14.3% identified the “need for large electronic health records or claims-based datasets” as a “great deal” ofa barrier.	*“One of my major issues… is data access. The processes change all the time.” – Participant 21, FG 4*	**Triangulation:** Both data sources pointed to inconsistent data access as a barrier, indicating a need for a standardized, centralized data management system.
Space for research	11.8% reported limited access to research space and infrastructure as “a great deal to quite a bit” of abarrier.	*“If we have funding, then lab space should immediately come.” – Participant 15, FG 3*	**Explanatory:** The qualitative data provided more context to the quantitative findings, highlighting that even when funding is secured, the lack of immediate access to infrastructure remains a significant barrier to conducting research.
**Needs**			
Research networking	34% indicated a likelihood of using research networking resources within the next 6 months.	*“I had to go outside of this campus because I didn’t know who on campus did this, and so I ended up kind of just going outside because there wasn’t anywhere I could just type in “patient-reported outcomes” and see who came up.” – Participant 6, FG 1*	**Convergence:** Both data types converged to highlight the need for web-based research networking.
Scientific writing support	46% likely to use scientific writing resources in the next 6 months.	*“So, like it would be nice if someone who had a lot of experience in other funding mechanisms and can review the contents to make sure everything is tight, like the science and budgets.” – Participant 19, FG 4*	**Expansion:** Quantitative data revealed a high interest in scientific writing support, while qualitative data expanded on this need, emphasizing the role of mentorship and advocacy in research success.
Access to local data sources	39% showed interest in accessing local data sources and research facilities.	*“We don’t see a central place where we can go for help. It’s very difficult to build a case if we can’t get to the data we are generating here.” – Participant 3, FG 1*	**Explanatory**: Qualitative data explained the survey findings by highlighting the difficulty in locating data resources, indicating a need for centralized data access points.
More pilot funding	*“I think we need more bridge funding. For instance, if you’ve applied for a federal grant but haven’t secured it, there should be more opportunities to bridge that gap. Ideally, there would be an infrastructure—a think tank—to strategize collectively. While we all do this individually, having additional resources at the campus level would be beneficial. It’s about moving everyone forward so that next time, as a campus, we are successful.”– Participant 9, FG 2*		**Expansion:** Qualitative data expanded on the survey findings by underscoring the need for bridge funding. Participants pointed out the difficulties in sustaining momentum after unsuccessful federal grant applications and highlighted the value of campus-level support and think tank resources to bridge these funding gaps.
Access to trained mentors	*“I think this is what OSCTR can maybe help with. Like, what’s your trajectory? What do you need to do? This can be done by anybody, anybody senior to us. People that have been successful in their academic careers can provide mentorship, career mentorship.” – Participant 14, FG 2*		**Expansion:** Qualitative data expanded on the survey findings by emphasizing the importance of access to trained mentors. Participants highlighted senior academics who had succeeded as valuable sources for mentorship and career advice.
**Facilitators**			
Professional development	Clinical study design (43%), data analysis (43%), and scientific writing (46%) resources provided by OSCTR were identified as facilitators.	*“Attending the training workshops helped refine my study design skills, which I can now apply directly to my ongoing projects.” – Participant 7, FG 1*	**Convergence:** Both data sources highlighted the importance of professional development, suggesting that continued training in these areas could significantly boost research capabilities.
Mentoring	30% found mentoring programs provided by OSCTR to help advance their research.	*“My department chair has been incredibly supportive—not just with what I need to do my job, but also career advice and general guidance. I have a few mentors among my colleagues who essentially trained me to do the basic research I’m doing now. Clinical research felt like diving into the abyss, but I was fortunate to accidentally find a couple of great mentors who have been invaluable.” – Participant 14, FG 3*	**Complementarity:** Quantitative data indicated the benefit of mentoring, while qualitative insights highlighted the need for structured, consistent mentorship programs for greater impact.
Bench-to-bedside collaboration	35% reported that bench-to-bedside collaboration activities facilitated by OSCTR have advanced their research.	*“Our collaborative meetings with clinical teams have accelerated our translational research, turning lab results into patient care practices.” – Participant 6, FG 1*	**Explanatory:** The qualitative data explained the quantitative interest in collaboration and highlighted the value of these collaborations.
Data management	33% utilized data management resources from OSCTR, finding them beneficial.	*“Having access to a central database allowed us to identify cohorts more efficiently, streamlining our recruitment process.” Participant 3, FG 1*	**Triangulation:** Both the quantitative and qualitative findings pointed to data management as a crucial area, suggesting that targeted training could address a widespread challenge in research.

OSCTR = Oklahoma Shared Clinical and Translational Resources.

## Discussion

This study provides a descriptive analysis of the barriers, needs, and facilitators affecting CTR in Oklahoma. By integrating quantitative (survey) and qualitative (focus group) data, our mixed-methods approach allowed an in-depth exploration of the key factors shaping the research environment. This approach not only identified broad trends through the survey but also provided depth and a detailed understanding of these factors through qualitative interviews. Additionally, our study highlights the successes and ongoing challenges in the research landscape. While OSCTR’s significant investment in infrastructure and resources has positively contributed to CTR in Oklahoma, the barriers, needs, and facilitators identified in this study offer essential perspectives on areas where further alignment between institutional structures and the evolving needs of clinical research could further enhance research productivity and support.

### Barriers

One key barrier identified through convergent meta-inference was the lack of protected time for research, an issue consistently highlighted in both the survey and focus group data. Over 70% of participants identified as clinical researchers, and both data types underscored the challenge of balancing patient care with research responsibilities. The dual demands of conducting patient-oriented research while managing teaching, administrative, and clinical duties emerged as a key hindrance to research productivity. This convergence reinforces existing literature identifying protected time as a critical factor in enabling research productivity and career advancement [16,17]. Researchers involved in clinical studies often face unique challenges balancing their research projects with other obligations, which can dilute their focus and impede progress [18–20]. Without sufficient protected time, researchers may experience increased stress and reduced output, ultimately impacting both individual success and the broader research capacity of their institutions [16–21]. For instance, Elias et al. reported that the absence of protected time significantly contributes to reduced productivity and job satisfaction among faculty in academic settings [17]. This aligns with our findings, where clinical researchers expressed that competing professional demands compromised their ability to engage meaningfully in research. Protected time is not just a facilitator of productivity but a fundamental requirement for high-quality research, particularly in translational research fields where sustained focus and dedicated effort are essential [17,22]. Studies have also shown that strategies such as flexible scheduling, course buyouts, and administrative support for grant management can help mitigate the pressure on clinician-researchers, allowing them to allocate adequate time to research [17,22]. These interventions not only foster a conducive environment for research but also contribute to better career progression and higher retention rates among clinician-researchers [23].

Funding emerged as a complementary theme. While survey data rated pilot and bridge funding as moderate barriers, qualitative narratives revealed their critical role in sustaining momentum between funding cycles and supporting collaborative research. Participants emphasized the challenge of building and retaining research teams without predictable internal support. These findings highlight the need for institutional investments that provide flexible funding across the research continuum, particularly during early-stage development and transitional periods [12,18,24]. To address this, institutions might consider expanding internal programs offering interim funding during critical gaps and establishing centralized think tanks or advisory bodies to support strategic grant planning and continuity [16,18,24–27]. Additionally, targeted initiatives tailored to collaborative or interdisciplinary research could better meet the evolving needs of the CTR workforce [25].

Administrative and regulatory challenges, such as IRB processes and inter-institutional coordination, were highlighted through explanatory meta-inference. Though less prominent in survey responses, focus group accounts painted a picture of institutional inefficiencies, delays, and communication breakdowns that impeded research progress. These findings clarify the quantitative ratings and suggest that even moderate administrative burdens may have an outsized impact on workflow and morale, supporting recommendations for streamlined IRB processes and enhanced inter-institutional agreements [18,28]. Issues related to data access showed strong triangulation between survey and focus group responses. Researchers reported difficulties locating and gaining access to large datasets and noted the lack of a centralized data management system. Both data strands underscore the importance of standardizing data processes and creating shared repositories to improve research efficiency and reproducibility [29,30]. The consistency of these findings across methods strengthens the argument for data infrastructure reform. Finally, the challenge of accessing physical infrastructure, such as lab and meeting spaces, emerged through explanatory inference. Although considered a lower barrier in the survey, qualitative data showed that these logistical delays often occurred even after funding was secured, disrupting research timelines. This aligns with existing research showing how misalignment between funding and facility readiness can create logistical delays and reduce institutional research output [31]. Addressing these gaps through proactive planning and resource allocation could improve efficiency and create a more supportive research environment.

### Needs

Research networking emerged as a top priority across both data strands, showing clear convergence. Quantitatively, over one-third of participants indicated a likelihood of using such resources in the next six months, and qualitatively, researchers described the challenge of identifying collaborators, sometimes seeking partnerships outside their institutions due to the absence of centralized directories. This alignment highlights the institutional value of searchable databases or online platforms to promote collaboration and interdisciplinary science, consistent with recommendations in the literature [32,33].

The need for scientific writing support reflected a pattern of expansion. While survey data indicated broad interest in writing assistance, qualitative data emphasized the added need for mentorship and expert feedback on proposal and manuscript development. Participants shared how peer support, access to experienced grant writers, and mentorship could enhance research quality and competitiveness. These findings highlight the importance of structured writing programs, review groups, and senior-to-junior mentoring models that support technical writing and provide strategic guidance for securing funding and publishing high-impact work [34–36].

Access to local data sources and facilities was another commonly reported need, demonstrating explanatory meta-inference. While nearly 40% of survey respondents expressed interest in these resources, focus group narratives clarified systemic barriers that hinder effective use, such as the lack of awareness about data custodians or outdated protocols. These findings suggest that addressing this need requires more than access**,** it calls for centralized, well-maintained portals and consistent institutional communication to facilitate data utilization [33,36–38].

Notably, two needs emerged exclusively from qualitative data, showing expansion not captured in survey responses: the need for bridge/pilot funding and access to trained mentors. Participants emphasized that gaps in support during unfunded periods or post-submission phases can halt momentum and lead to burnout or attrition, particularly for early-career investigators. Similarly, they identified the absence of formal mentorship structures as a critical gap, noting that while some found informal guidance, many lacked reliable access to mentors who could support career development, grant navigation, and long-term planning [19,35,39–41]. These findings reinforce the need for sustained investment in bridge funding mechanisms and structured, goal-oriented mentoring programs to promote equity in research success and retention [12,18,42–45].

### Facilitators

The findings from this study highlight several key facilitators of CTR, with strong support from both data strands and primarily demonstrating convergence, complementarity, and triangulation. The role of professional development, including training in clinical study design, data analysis, and scientific writing, exhibited apparent convergence between survey data and focus group responses. Participants described how OSCTR’s workshops enhanced their research skills and increased their confidence in pursuing new studies. This dual support affirms that institutional training programs are appreciated and impactful and that expanding these offerings, especially for early-career or researchers from underrepresented backgrounds, can strengthen institutional research capacity [18,46]. Bench-to-bedside collaboration also showed a meaningful explanatory relationship. Participants described how collaborative meetings with clinical teams accelerated translation from lab to practice. This finding highlights the importance of institutional structures supporting translational dialog, as researchers can achieve greater real-world impact. This is a critical aim of the CTR initiative, bridging the gap between basic science and clinical application [7,47]. Finally, data management resources were identified through triangulation. Quantitative data showed an uptake of OSCTR’s data management support, while qualitative accounts emphasized how centralized databases enhanced recruitment efficiency and study planning. This alignment suggests that investment in accessible data infrastructure and training can address a recognized need and significantly streamline research workflows [48].

## Implications for practice

While some of the challenges identified in this study, such as limited protected time, mentorship needs, and grant support, are common across research environments, several findings appear uniquely specific to Oklahoma. Participants repeatedly emphasized the lack of centralized infrastructure for research collaboration and data access, reporting challenges in identifying collaborators and navigating fragmented or inconsistent data-sharing protocols. These challenges are compounded by Oklahoma’s geographic dispersion and institutional silos, spanning urban medical centers, rural health systems, and sovereign tribal nations, each with differing access to research infrastructure and support. Additionally, participants described a heavy reliance on OSCTR as the primary CTR support mechanism, with few alternative institutional resources available. This overcentralization highlights the need for decentralized, regionally responsive support systems, including local mentorship hubs, institution-specific pilot funding, and interoperable data platforms. Participants also pointed to limited cross-institutional coordination, which hindered interdisciplinary collaboration and created duplicative efforts across campuses. Tailoring solutions to these state-specific realities will be critical to strengthening the research environment and improving health outcomes in Oklahoma.

Our study findings offer actionable suggestions for enhancing the CTR environment in Oklahoma, which can inform the continued development of the OSCTR, recently renewed for another 10 years, as well as other similar mechanisms nationwide. Interventions such as implementing structured mentoring programs, streamlining administrative processes, and creating centralized data management systems could address the barriers and needs identified in this study [27,35,41,42]. Additionally, innovative communication strategies, such as podcasts and newsletters, can help connect researchers to available resources and foster collaboration [49]. A prime example is the *GROklahoma Podcast*, launched in 2023 by OSCTR in response to reported gaps in awareness and researcher isolation [50]. The podcast highlights available research resources, funding opportunities, and interviews with CTR scientists and support staff, fostering greater awareness and collaboration among researchers across the state. This locally developed platform exemplifies how Oklahoma’s CTR leaders are using novel, low-barrier tools to bridge geographic and institutional divides. Such an approach may serve as a model for other resource-constrained states.

OSCTR’s renewed funding also presents a timely opportunity to expand pilot and bridge funding mechanisms, strengthen mentorship infrastructure, and enhance professional development workshops, particularly those tailored to rural, tribal, and early-career investigators. As Oklahoma’s CTR landscape continues to evolve, these findings should guide strategic investments across the state and inform national conversations on sustaining equitable research environments in low-capacity settings. Based on our mixed-methods findings, we identified several priority areas for improving CTR infrastructure and support across Oklahoma. These recommendations may serve as a roadmap for targeted investment in state-specific research infrastructure and collaborative strategies. A summary of these priorities is presented in Table 3.

**Table 3.** Recommendations to strengthen clinical and translational research infrastructure in Oklahoma

Category	Recommendation
Decentralized support	Establish regionally responsive CTR support systems, including local mentorship hubs and locally accessible funding mechanisms.
Funding sustainability	Expand pilot and bridge funding opportunities to sustainresearch momentum, particularly during gaps between grant cycles.
Data access and sharing	Develop centralized, searchable data platforms and streamline data-sharing protocols across institutions, including tribal, rural, and VA systems.
Mentorship infrastructure	Established structured mentorship programs that connect early-career investigators with experienced investigatorsto support career development and grant success
Collaboration and networking	Create institutional platforms for research networking, enabling collaboration across disciplines and organizations statewide.
Targeted professional development	Enhance professional development offerings in study design, scientific writing, and data analysis, tailored to the needs of rural and early-career researchers.
Administrative streamlining	Streamline IRB and administrative processes to reduce bottlenecks and support multi-institutional research initiatives.
Communication and awareness	Leverage innovative communication tools, such as podcasts and newsletters, to increase the visibility of available resources, build a stronger statewide research community, and reduce researcher isolation.

*Note:* Recommendations are based on integrated findings from surveys and focus groups using convergence, complementarity, and explanatory integration across data strands with clinical and translational research stakeholders in Oklahoma. CTR = clinical and translational research.

## Strengths and limitations

This study comprehensively examined the barriers, needs, and facilitators to conducting CTR in Oklahoma using a sequential, descriptive mixed-methods approach. A key strength of this study lies in its integration of quantitative and qualitative data, which provided both a broad understanding of trends and a deep exploration of individual experiences. This design also enabled a nuanced perspective of the CTR landscape across the state. Focusing on Oklahoma offered important regional insights, especially regarding the structural and geographic challenges that researchers face in rural, tribal, and underserved settings. Additionally, by targeting experienced Principal Investigators (PIs) and Co-Investigators (co-Is), the study highlights the challenges at the leadership level of research teams, providing actionable recommendations for enhancing institutional support and research productivity.

Nonetheless, several limitations warrant consideration. First, while informative, the sample size may not fully capture the diversity of perspectives across all research roles, particularly for the qualitative component. Focusing on PIs and Co-Is may have excluded early-career researchers, trainees, or staff facing CTR-related challenges. Second, the time gap between the survey (2019) and focus group data collection (2020–2022), compounded by the COVID-19 pandemic, may have introduced variability due to shifts in institutional priorities and research demands. Additionally, the ongoing impact of the pandemic likely contributed to evolving research priorities and institutional adaptations, which may have influenced participants’ experiences during this period. Despite these limitations, this study provides a robust foundation for institutional and statewide efforts to strengthen CTR in Oklahoma and similarly situated research environments.

## Conclusion

This mixed-methods study offers a comprehensive assessment of the CTR landscape in Oklahoma, identifying key barriers, needs, and facilitators that shape research participation and productivity. The convergence of findings across data strands, particularly regarding protected research time, pilot funding, mentorship, and collaboration, highlights their centrality to advancing CTR efforts. Qualitative data expanded on these findings by contextualizing the institutional and interpersonal dynamics that influence research engagement and success. Tailored interventions such as structured mentoring programs, centralized data access, enhanced research networking platforms, and streamlined administrative processes are essential for fostering a more supportive research ecosystem.

Advancing CTR in Oklahoma is essential for addressing persistent healthcare challenges, promoting health equity, and improving patient outcomes across diverse populations. By investing in research infrastructure, encouraging interdisciplinary collaboration, and cultivating strong institutional and community partnerships, Oklahoma can strengthen its research capacity and build a more resilient and responsive research environment. A summary of context-specific recommendations to guide these efforts is presented in Table 3, offering a practical roadmap to inform institutional strategy, resource allocation, and statewide coordination.

Importantly, Oklahoma’s experience offers a transferable model for other states confronting similar geographic, structural, and capacity-related challenges. Strengthening CTR is vital for scientific advancement and a necessary step toward realizing equitable healthcare solutions and improving the well-being of all Oklahomans.

## Supporting information

10.1017/cts.2025.10066.sm001 Ogunsanya et al. supplementary material 1 Ogunsanya et al. supplementary material

10.1017/cts.2025.10066.sm002 Ogunsanya et al. supplementary material 2 Ogunsanya et al. supplementary material

## Supplementary material

The supplementary material for this article can be found at https://doi.org/10.1017/cts.2025.10066
