# Supplementary material for: Exploring barriers, needs, and facilitators for clinical and translational research in Oklahoma: A sequential mixed-methods study
Source: J Clin Transl Sci. 2025 Jun 18;9(1):e155. doi: 10.1017/cts.2025.10066 (PMC12392360; doi:10.1017/cts.2025.10066)
Supplement: Ogunsanya et al. supplementary material 1 — Ogunsanya et al. supplementary material [file S2059866125100666sup001.docx]

**OSCTR Needs Assessment Survey**

INTRODUCTION AND CONSENT

Your participation in this survey is completely voluntary. Your responses will be kept strictly confidential. Only the Tracking and Evaluation Core within OSCTR will have access to individual responses. Also, if you are willing to participate in a follow-up interview so that we can further explore CTR needs and barriers, we ask that you provide your name and contact information at the end of the survey. All of your responses will be kept completely confidential.

If you have any questions about this survey, you may contact Dr. Laura Beebe, Director of Tracking and Evaluation, by phone at 405-271-2229 ext. 48061 or by e-mail at laura-beebe@ouhsc.edu.

If you would like more information about OSCTR services, programs and resources, contact the OSCTR at 405-271-3480 or by e-mail at OSCTR@ouhsc.edu.

DESCRIPTION OF RESEARCH ACTIVITIES

What TYPE of research do you do? Check all that apply.

- Basic Research
- Clinical (or patient-oriented) Research
- Public or Population Health Research
- Education Research
- Engineering Research
- Community-Based Participatory Research
- Health Services Research
- Methodological Research
- Dissemination and Implementation Research
- Other (please specify) ___________________

BARRIERS

In the past year, to what extent have you experienced barriers or challenges with the following:

|  | Not at all | Very little | Somewhat | Quite a bit | A great deal | N/A |
| --- | --- | --- | --- | --- | --- | --- |
| Proposal development |  |  |  |  |  |  |
| Statistical design |  |  |  |  |  |  |
| Needs for large Electronic Health Record (EHR) or claims based data sets |  |  |  |  |  |  |
| Data analysis |  |  |  |  |  |  |
| IRB inter-institutional collaboration |  |  |  |  |  |  |
| Protected time for research |  |  |  |  |  |  |
| Pilot project funding |  |  |  |  |  |  |
| Study participant recruitment |  |  |  |  |  |  |
| Grant administration |  |  |  |  |  |  |
| Space for research |  |  |  |  |  |  |
| Shared instrumentation |  |  |  |  |  |  |
| Regulatory issues |  |  |  |  |  |  |
| Commercial development |  |  |  |  |  |  |
| Compliance with the NIH Open Access Policy |  |  |  |  |  |  |
| Citing grant support in manuscripts |  |  |  |  |  |  |
| Converting an abstract presented at a professional meeting into a manuscript for publication |  |  |  |  |  |  |

Have you experienced other barriers to conducting research? Yes No

Please describe: _____________________

What do you find is your greatest barrier to performing clinical and translational research? ___________

How satisfied are you with your institution’s overall efforts at supporting clinical and translational research?

Not at all satisfied

Slightly satisfied

Moderately satisfied

Very satisfied

Extremely satisfied

NEEDS: OSCTR SERVICES AND RESOURCES

The OSCTR aims to provide a wide range of services to support CTR. Please rate your level of interest in using the following resources:

|  | Likely to use in next 6 months | Not likely to use in next 6 months | I am not sure what this is | I would like more information |
| --- | --- | --- | --- | --- |
| PROFILES web-based research networking |  |  |  |  |
| Online tutorials, flowcharts, and templates for regulatory/institutional processes, grants, and manuscripts |  |  |  |  |
| Individual assistance with policies/procedures and accessing research services |  |  |  |  |
| Assistance with IRB/regulatory processes |  |  |  |  |
| Recruiting participants for research studies |  |  |  |  |
| Research-related clinic visits and clinical services |  |  |  |  |
| Clinical testing equipment |  |  |  |  |
| Clinical research personnel |  |  |  |  |
| Research registries and repositories |  |  |  |  |
| Biospecimen processing and storage |  |  |  |  |
| Data analysis expertise |  |  |  |  |
| Study design expertise |  |  |  |  |
| Survey research expertise |  |  |  |  |
| Survey data collection |  |  |  |  |
| Building and managing research databases |  |  |  |  |
| Qualitative data collection |  |  |  |  |
| Community engagement: working with special populations |  |  |  |  |
| Scientific writing: grant preparation |  |  |  |  |
| Scientific writing: manuscript preparation |  |  |  |  |
| Mentor/mentee training |  |  |  |  |
| Using iLab to access core facilities |  |  |  |  |

What additional services would you like to see provided by the OSCTR?__________________________

**Facilitators: Professional development and research activities**

The OSCTR Professional Development Core provides educational opportunities to foster CTR capabilities of investigators. Which of the following CTR-related activities provided by OSCTR has helped you the most in advancing your research?

Bench to bedside collaboration

Bedside to community translational research

Collaboration with industry partners

Best practices for mentoring

Mentoring under-represented groups

Data security

Recruitment and retention of study participants

Monitoring protocol adherence

Designing clinical studies

Data analysis for clinical studies

Data safety monitoring

Electronic data for cohort identification

Bioinformatics tools

mHealth technology in research

Scientific writing

Presentation skills

Accessing local data sources and research facilities

Other (specify other)_____________________________

OVERALL SATISFACTION

How satisfied are you with your institution's overall efforts at supporting clinical and translational research?

- Not at all
- Slightly
- Moderately
- Very
- Extremely

PROFESSIONAL BACKGROUND

What is your PRIMARY College affiliation? Please select from the following drop-down menus.

OUHSC-College of Allied Health

OUHSC-College of Dentistry

OUHSC-College of Medicine

OUHSC-College of Nursing

OUHSC-College of Pharmacy

OUHSC-College of Public Health

OU Tulsa campus

What is your current position or academic **rank**?

Post-doctoral/clinical fellow

Instructor

Assistant Professor

Associate Professor

Professor

Staff

Other (please specify) _____________________

What degrees do you hold? Check all that apply.

AuD MA MSN

DDS MBA MSW

DNSc MD NP

DO MLIS PA

DrPH MPH PharmD

DVM MS PhD

EdD MSEd ScD

JD

Other (please specify) ________________

What is your gender? Male Female Prefer not to answer

If you would be willing to participate in a follow-up interview about your experience with OSCTR or needs related to CTR, please provide your name and contact information below.  If you are selected to participate, you will be contacted with additional details. Be assured that your identity will be kept strictly confidential.

- Name (1) ________________________________________________
- E-mail (2) ________________________________________________
- Preferred Phone Number (3) ________________________________________________

END OF SURVEY

Thank you for taking the survey.

**If you would like to speak with someone to discuss what OSCTR can do to facilitate your Clinical Translational Research or to get more involved in CTR, please call 405-271-3480 or email OSCTR@ouhsc.edu.**
