# Supplementary material for: Exploring barriers, needs, and facilitators for clinical and translational research in Oklahoma: A sequential mixed-methods study
Source: J Clin Transl Sci. 2025 Jun 18;9(1):e155. doi: 10.1017/cts.2025.10066 (PMC12392360; doi:10.1017/cts.2025.10066)
Supplement: Ogunsanya et al. supplementary material 2 — Ogunsanya et al. supplementary material [file S2059866125100666sup002.docx]

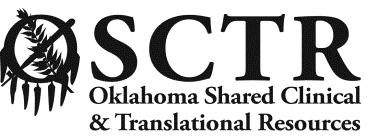

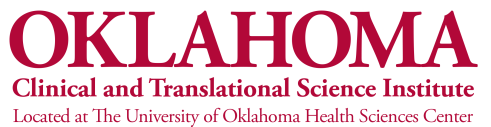


**OSCTR NEEDS ASSESSMENT**

**Focus Group Moderator’s Guide**

**Moderator: Motolani Ogunsanya, PhD**

**Assistant Moderator: Nicole Holmes**

## Introduction:

Good afternoon and welcome to our focus group discussion. This is a follow-up to the recent needs assessment survey that you participated in. We’re here to learn more about barriers and needs around conducting clinical and translational research, as well as potential solutions and how best to implement these solutions. By identifying these common needs and barriers experienced, we would like to build some programs or infrastructure or tools to bridge the gaps or to eliminate some of the issues that are being experienced.

My name is Motolani Ogunsanya (you may call me Mo!); I represent the OSCTR Evaluation Core and I will serve as the moderator and scribe for today’s focus group discussion. Assisting me today is our OSCTR evaluation coordinator, Nicole Holmes.

The purpose of today’s discussion is to tap into your thoughts and opinions regarding barriers you have experienced with performing clinical and translational research. We would like you to think about your experience as a whole – everything from regulatory and administrative processes, to issues locating resources, to not having enough protected time for your research. The purpose of this focus group is to identify barriers and to find solutions for overcoming them. We hope to be able to use the information we collect from you today during this discussion to implement processes or resources to prevent these barriers from hindering your research.

This focus group discussion will take no more than **90 minutes**. This discussion will be tape recorded so we don’t miss any of your comments and Nicole will be taking notes, as well. We have name tents here in front of us to help me remember names, but no names will be included in any reports as we will report our findings in aggregates. All of your comments will remain confidential and will not be used as part of your job evaluation or in any implicative manner.

If you have a cell phone, please put it on the quiet mode, and if you need to answer, step out to do so. Feel free to get up and get more refreshments or use the bathroom if you like.

## Ground Rules:

Let’s discuss a few ground rules. As the session moderator, I will ask the questions and keep everyone on track. I will keep track of time, and therefore, I may need to interrupt the discussion to move forward in the interest of time. It is important that everyone feels comfortable and at ease during the discussion. There are no right or wrong answers to the questions I am about to ask. We expect that you will have differing points of view. Please feel free to share your point of view even if it is different from what others may have said. If you want to follow up on something someone has said, you want to agree, disagree, or give an example, feel free to do that. I am here to ask questions, listen and make sure everyone has a chance to share. We’re interested in hearing from each of you and we want to make sure everyone has a chance to share their ideas.

| We Want You To Do The Talking.  - We would like everyone to participate. - Only one person speaks at a time. - You do not have to speak in any particular order. - I may call on you if you if I haven’t heard from you in a while.  There Are No Right or Wrong Answers.  - Everyone’s experiences and opinions are important. - You do not have to agree with the views of other people in the group. - We want to hear a wide range of opinions.  1. **What Is Said In This Room Stays Here.**  - We want everyone to feel comfortable sharing information.  1. **We Will Be Tape Recording The Group.**  - We want to capture everything you have to say. - We won’t identify anyone by name in our report. - You will remain anonymous. |
| --- |

**Opening Activity:**

Let’s begin. Let’s find out more about each other by going around the table. (Ask an ice-breaker question about dream vacation or plans for the next holiday).

1. Next, tell us about your research area of expertise.
2. What type of research do you do?

***Post the flip chart page on the wall.***

***Call on each participant around the table.***

***Write the data on the flip chart.***

**Transitional Question:**

1. ***Reasons for participating in this focus group.***

Ok. Let’s move on. We would like to know what made you decide to attend this focus group today.

**Probes:**

- What made you provide your contact information in the survey?
- What prompted you to participate?

Why was this important to you?

- What made you decide to provide your contact information?
- What prompted you to participate?
- Why was this important to you?

**Additional Probes/Examples:**

- To improve the research experience?
- To improve barriers related clinical and translational research
- Networking?
- Something else?

***Call on each participant around the table.***

***Write responses on the flip chart.***

***If several participants nod in agreeance or voice the same opinion, ask for a show of hands.***

**Key Questions (based on survey findings):**

1. ***Needs and Barriers to CTR***

Let’s talk about barriers.

- Many barriers were identified from the needs assessment survey. We will talk about these barriers in more detail a little later but first let’s make a list of barriers you have personally experienced with performing CTR.
- You will see paper and pens on the table in front of you. Please take a couple of minutes to jot down some of the barriers you have experienced. Whatever comes to mind?
- *Pause/give time for them to make their lists.*
- Okay now let’s talk about your lists. What were some of the barriers you listed? Just feel free to shout them out, let’s list them on the flip chart.

***Call on participants at random from around the table. If there’s someone you haven’t heard from or that has been particularly quiet, call on them. Write responses on the flip chart. If several participants nod in agreeance or voice the same opinion, ask for a show of hands.***

- Okay now let’s get a little more specific. What was the greatest barrier you faced to performing clinical and translational research? Go ahead and shout them out.

***Call on participants at random from around the table. If there’s someone you haven’t heard from or that has been particularly quiet, call on them. Write responses on the flip chart. If several participants nod in agreeance or voice the same opinion, ask for a show of hands.***

- Go ahead and write the greatest barrier in the box on your sheet. Thank you. Once you are finished, Nicole will collect your papers.
- Post the listing of barriers the group just identified on the wall. Next, hand out a list of barriers that were identified from the needs assessment survey.
- Nicole is handing out a list of the common barriers identified in the survey. We’re going to talk about these a little more in depth.

**Probes/Examples (generated from survey):**

- Resources
  - Identifying resources, funding opportunities, implementation and dissemination
- Regulatory and administrative processes
  - Bureaucracy, IRB processes, ORA and contracting.
- Protected time
  - Honoring protected time, allocating time to do research, aligning departmental needs with professional aspirations.
- Professional collaboration
  - Intra-university. inter-university, mentorship
  - Teambuilding/campus events geared at getting to know others
- Lack of support
  - Institutional, administrative, research
  - Funding

1. **Needs/OSCTR Services**
   - Now let’s talk about needs. Think about your current needs but also about things you have struggled with in the past – be it locating resources, finding funding sources, assistance with recruitment, etc.
   - What can we do to help you perform CTR? What would make your life easier?

***Call on participants at random from around the table. If there’s someone you haven’t heard from or that has been particularly quiet, call on them.***

***Write responses on the flip chart.***

***If several participants nod in agreeance or voice the same opinion, ask for a show of hands.***

- - What would make your research easier? What needs to you have?
  - What do you think would be helpful for new researchers in the area?
  - Let’s discuss some of the needs that were identified from the needs assessment survey. We would like to get more insight from you regarding some of these needs.

**Probes/Examples (generated from survey):**

- Resources
  - Identifying resources, identifying potential collaborators, implementation and dissemination
- Training
  - Statistical analyses, grant writing, ABCs of OSCTR, teaching methods (up-to-date pedagogical methods), IRB
- Funding
  - Intramural and extramural grants
- Professional development
  - Mentorship (expand on level and quality)
  - Research support

1. **Now that we have talked about barriers and needs, let’s talk about potential solutions AND how best to implement these solutions. Thinking back to what you or someone said about barriers and needs, what are some of the ways to address these issues.**

**Probes/Examples:**

- Things that would make performing research easier
- Access to research coordinators/recruitment
- Assistance obtaining funding
- Assistance with manuscript preparation

1. **How best do we go about implementing these suggested solutions (if possible, list out the suggested solutions)**

**Additional Questions**

- What has contributed to your success?
- As a researcher and faculty member, what would you need now to be successful in your role?
- How would you describe the **level and quality** of support and guidance you receive here now?

**Closing Question:**

Is there anything that we missed or that that you came wanting to say that you didn’t get a chance to say?

**Conclusion:**

Thank you all so much for participating in our focus group. This was a wonderful discussion. Your opinions will be a valuable asset to the improvement of these issues we have discussed and identified today. We hope you have found the discussion interesting! If there is anything you are unhappy with or wish to give feedback about, please feel free to contact program staff or you are welcome to speak to me later. I would also like to remind you that any comments featured in our report will be anonymous.
